# Influence of the Gap between Substrates in the Laser-Induced Transference of High-Viscosity Pastes

**DOI:** 10.3390/ma14195567

**Published:** 2021-09-25

**Authors:** Juan José Moreno-Labella, David Munoz-Martin, Guillermo Vallejo, Carlos Molpeceres, Miguel Morales

**Affiliations:** 1 Centro Láser, Universidad Politécnica de Madrid, Alan Turing 1, 28038 Madrid, Spain; david.munoz@upm.es (D.M.-M.); carlos.molpeceres@upm.es (C.M.); miguel.morales@upm.es (M.M.); 2 Departamento de Física Aplicada e Ingeniería de Materiales, Escuela Técnica Superior de Ingenieros Industriales, José Gutiérrez Abascal 2, 28006 Madrid, Spain; guillermo.vallejo.pascual@gmail.com; 3 Departamento de Ingeniería Mecánica, Escuela Técnica Superior de Ingeniería y Diseño Industrial, Química y Diseño Industrial, Ronda de Valencia 3, 28012 Madrid, Spain

**Keywords:** laser-induced forward transfer (LIFT), laser process modeling, fluid dynamics, phase-field, laser direct write

## Abstract

Laser-induced forward transfer for high-viscosity—of Pa·s—pastes differ from standard LIFT processes in its dynamics. In most techniques, the transference after setting a great gap does not modify the shape acquired by the fluid, so it stretches until it breaks into droplets. In contrast, there is no transferred material when the gap is bigger than three times the paste thickness in LIFT for high-viscosity pastes, and only a spray is observed on the acceptor using this configuration. In this work, the dynamics of the paste have been studied using a finite-element model in COMSOL Multiphysics, and the behavior of the paste varying the gap between the donor and the acceptor substrates has also been modeled. The paste bursts for great gaps, but it is confined when the acceptor is placed close enough. The obtained simulations have been compared with a previous work, in which the paste structures were photographed. The analysis of the simulations in terms of speed allows for predicting the burst of the paste—spray regime—and the construction of a printability map regarding the gap between the substrates.

## 1. Introduction

Laser-induced forward transfer (LIFT) techniques are a group of processes for the transference of a great variety of materials, such as biological fluids, metallic inks and pastes, or solid pellets of material [1]. In them, the contact between the printing elements and the substrate is avoided, removing the chance of nozzle clogging in the process. Their great design flexibility, the absence of a prebuilt pattern, and their capability of transferring a small amount of material [2] are their main advantages when compared to other direct-write techniques, such as stamping or molding [3].

**Figure 1:**
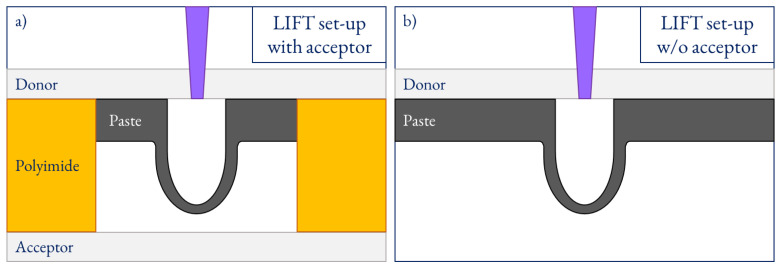
LIFT setup for the transference of high-viscosity pastes with an acceptor at a gap set by a polyimide tape (**a**) and without acceptor (**b**).

In most LIFT variations, the fluid is spread directly onto a transparent-to-the-laser donor substrate (Figure 1a). When the laser interacts with the fluid, a high-pressure bubble appears, whose dynamics eject a portion of the fluid towards an acceptor substrate [4], placed at a distance far from the donor—the gap (Figure 1b). The interaction between the laser and the material may harm the latter if its absorption is low enough to be considered volumetric, as in biofluids or water-based solutions. In the case of opaque materials to the selected laser wavelength, the absorption is shallow enough to be considered only superficial, and the possible harm is neglectable.

The case of LIFT for high-viscosity pastes implies several considerations that govern the process. Despite the setup and the push of the fluid due to the formation of a bubble being quite similar, the rheological properties of the paste make the dynamics of the process differ from that of LIFT for less viscous fluids. The expansion of the bubble towards the free surface needs to overcome the viscous forces, so it may be trapped in the paste if the laser pulse energy is not high enough. Furthermore, the less viscous fluids can refill the cavities generated by the laser in the donor substrate, whereas the pastes would partially preserve their shape after the transference. The absence of elastic behavior in the case of the transference of the pastes also makes the non-transference structures much more visible than in regular water-based LIFT fluids, as they remain for long times.

LIFT for high-viscosity pastes processes does not involve the generation of a transference jet, but instead has a bell-like shape due to the expansion of the small amount of vaporized material [5,6]. The overexpansion of this bell-like structure makes it burst when the thickness of this paste husk is too low. The colloid nature of the pastes—built with a mixture of micrometer-sized metallic particles and an organic solvent—makes the rupture asymmetrical and random, in the shape of paste clusters instead of a splash of tiny droplets.

To simulate LIFT processes and their variants, three main approaches have already been published, namely the Level Set [7,8,9,10], Volume of Fluid [11], and Phase Field [12] methods. The modeling of high-viscosity pastes has not been faced until now though. In this work, a Phase Field model is proposed to study the shape of the inner bubble that turns up in metallic, high-viscosity pastes. The validation of the model is made using published data from a previous work of our research group [13] by replicating the different regimes of transference that were observed through an ultra-fast image acquisition system. Once validated, some effects observed in the images acquired using confocal microscopy are studied in the model, such as the final shape of the donor and the extreme interactions between the paste and the acceptor. In the images and the experiments, the gap and the paste thickness seem to be related, as the transference is much more difficult for big gaps or thin paste layers. The analysis of the simulations allows for studying this phenomenon and proposing a more specific criterion for this ratio.

## 2. Determination of the Material Properties

High-viscosity pastes are complex solutions, halfway between colloids and fluids. They are formed by suspending micron-size particles of a metallic material in an organic solvent (DuPont 9450, DuPont, Bristol, UK), so their proportion is critical to determine their properties. In this work, two commercial metallization silver pastes from DuPont were studied, under the commercial names of PV17F and PV19B.

**Figure 2:**
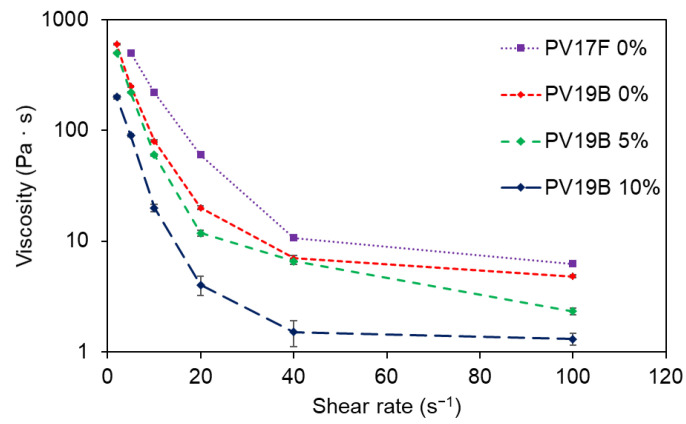
Viscosity against shear rate for DuPont^®^ PV17F and PV19B silver pastes considering different added solvent concentration mixtures: raw PV17F paste [14] (purple squares, dotted line), raw PV19B paste (red diamonds, short-dashed line), PV19B paste with 5 wt. % solvent concentration (green diamonds, medium-dashed line), and PV19B paste with 10 wt. % solvent concentration (navy diamonds, long-dashed line).

To characterize the rheological properties of the pastes, a Brookfield DV-II+ (Brookfield Engineering Laboratories Inc., Stoughton, MA, USA) viscosimeter was used. These pastes exhibited a remarkably non-Newtonian behavior, as shown in Figure 2. LIFT processes induce an extremely high shear rate, so the viscosities drop far below the values specified in the datasheet (280–400 Pa·s at 10 rpm for PV17F, and 130–250 Pa·s at 20 rpm for PV19B). This behavior is explained rheologically and not thermally, because the amount of vaporized paste is very small, and the heat conduction takes place at a slower time scale than the material transference [12]. Provided that they are not homogeneous materials but a suspension of particles, there is a wide range for the given viscosity. The study of the viscosity for both pastes considering different organic solvent proportions reports the data as shown in Figure 2. In the figure, the solvent percentage expresses the relative quantity of organic solvent that has been added to the commercial paste. In each paste, the solids content is around 90% of the total weight of the paste. The viscosity values on the technical sheets stand for low-shear-rate values at the specified revolutions per minute. The measured viscosity values, searching for the infinite viscosity, are far below the values in the technical datasheets.

## 3. Numerical Model

### 3.1. FEM-CFD COMSOL^®^ Model

An axisymmetric FEM CFD model was built in COMSOL Multiphysics^®^ 5.3a (COMSOL AB, Stockholm, Sweden) [15] to simulate the process. This symmetry condition eliminates some asymmetrical observed effects that differ from the ideal conditions, such as radial paste ruptures or setting the surfaces unevenly. A Phase-Field method was chosen to track the fluid interface. In the COMSOL Multiphysics^®^ definition of the method [15], Φ, the auxiliary function to follow the interface, varied between −1 (fluid) and 1 (air), so the interface could be interpreted as centered around 0. Despite high-viscosity pastes not being sorted as fluids, the motion of each phase in the model is ruled by the continuity equation for the incompressible flow (Equation (1)) and momentum equation (Equation (2)), derived from Navier-Stokes Equations [16,17,18,19].

(1) ∇·u=0

(2) $$\rho \partial u/\partial t+\rho \left(u\cdot \nabla \right)u=\nabla \cdot \left[-\mathrm{pI}+\mu \left(\nabla u+\nabla u^{T}\right)\right]+F_{\mathrm{st}}+F_{g}$$

The inclusion of gravity—the last term in Equation (2), $F_{g}$—has also been considered, as it may modify the conditions for transference in the simulation. Nevertheless, the analysis of the results leads to a slight difference almost only noticeable in the velocity field, so this last term is neglectable. The penultimate term of Equation (2) stands for surface tension ($F_{\mathrm{st}}$), implemented in COMSOL^®^ Phase-Field methods as follows [20]:

(3) $$F_{\mathrm{st}}=G\nabla \phi$$

where G is the chemical potential, defined as follows:

(4) $$G=\lambda \left(-\nabla^{2}\phi +\phi \left(\phi^{2}-1\right)/\varepsilon^{2}\right)$$

In this definition, λ quantifies the mixing energy density, and ε represents a capillary width related to the thickness of the interface. There are additional equations to track the position of the interface precisely, namely:

(5) $$\partial \phi /\partial t+\left(u\cdot \nabla \right)\phi =\nabla \cdot \gamma \lambda /\varepsilon^{2}\nabla \psi$$

(6) $$\psi =-\nabla \cdot \varepsilon^{2}\nabla \phi +\left(\phi^{2}-1\right)\phi$$

These quantities are interrelated with γ and σ through the following equations:

(7) $$\sigma =2\sqrt{2}/3\cdot \lambda /\varepsilon$$

(8) $$\gamma ={\chi \varepsilon }^{2}$$

χ parameter is the mobility tuning parameter, which rules the diffusion time in the interface, following the Cahn−Hilliard diffusion law [21]. The amount of each phase is computed as follows:

(9) $$V_{f}=\min\left(\max\left(\left(1+\phi \right)/2,\;0\right),1\right)$$

Properties of the materials turn out the weighted sum of those of each phase:

(10) $$\rho =V_{f}\rho_{2}+\left(1-V_{f}\right)\rho_{1}$$

(11) $$\mu =V_{f}\mu_{2}+\left(1-V_{f}\right)\mu_{1}$$

The mean curvature of the interface can be calculated as follows:

(12) κ=2(1+ϕ)(1−ϕ)·G/σ

### 3.2. Numerical Model for Pressure Function

**Figure 3:**
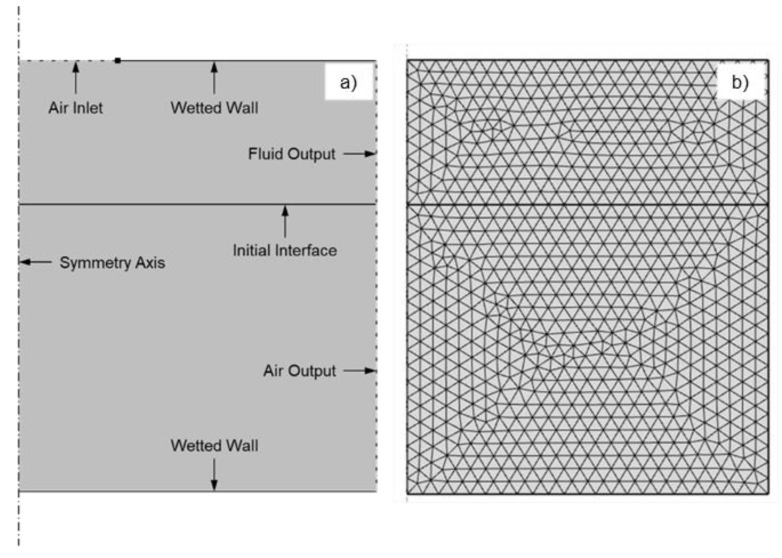
2D axisymmetric model sketch for the transference of high-viscosity pastes (**a**) and its mesh, which has been magnified to allow observing the elements inside (**b**).

In the model, the motion of the paste is caused by an inflow of air characterized by its pressure value—instead of a generation of a vaporized high-pressure area due to the interaction laser pulse-paste, as it happens experimentally. The analysis of the results in this work is given in terms of pressure, though this parameter is not typically used in LIFT studies, rather the laser pulse energy—either expressed in terms of irradiance, absolute energy, or fluence. Nevertheless, this relation between pressure and energy has been studied for confined interaction processes [22]. Considering that the pressure is proportional to the squared root of the laser irradiance, and applying the laser parameters to these approximations, the obtained pressure values are around hundreds of megapascals, which fits in the magnitude order of the values employed to simulate this sort of LIFT process. The movement of the paste is induced by an inflow of air on the top of the model that represents the vaporization due to the interaction between the laser pulse and the paste (Figure 3). Pressure evolution thus needs to be described as the model input. For this purpose, the pressure value has been estimated from the effects on the paste structures and from its comparison with the time-resolved images.

**Figure 4:**
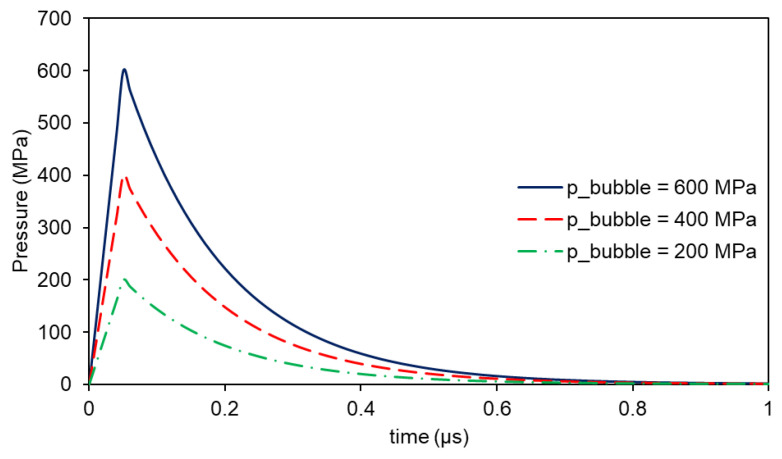
Pressure function considering a pressure peak of 600 MPa (blue solid line), 400 MPa (red dashed line), and 200 MPa (green dash-dotted line).

As the pressure model supposes only the interaction between the laser and the material and not the further expansion of the paste, the decays due to its movement and the thermal cooling should also be considered. The superposition of the obtained curve with the pressure decays leads to the pressure inlet function shown in Figure 4, taken as only being dependent on the pressure peak value. This peak value was estimated by observing the behavior of the paste and was not derived from the physical calculus under the model considerations. In the model, this piecewise function, defined as in Equation (13), was used as the only input, keeping the shape, and only varying the pressure peak value, p_bubble_. The maximum in time occurred at 0.05 μs—defined as t_max_—after a step-like function was defined, only for the smooth the transition between zero and pressure peak. The quickness of the decay could be adjusted by modifying τ.

(13) $$\{\begin{array}{cc} \mathrm{step}\left(t\right)\cdot p_{\mathrm{bubble}} & t<t_{\max}\\ p_{\mathrm{bubble}}\cdot e^{-\left(\frac{t-t_{\max}}{\tau }\right)} & t\geq t_{\max} \end{array}$$

In COMSOL Multiphysics, these pressure values are implemented as an inlet boundary, in which the pressure value, $p_{0}$, is defined as follows:

(14) $$n^{T}\left(-\mathrm{pI}+\mu \left(\nabla u+{\left(\nabla u\right)}^{T}\right)\right)n=-p_{0}$$

## 4. Model Validation

To model the metallic paste, a first attempt was made considering the Cross approach for non-Newtonian fluids. Nevertheless, the fluid suffered from such high shear rates during the LIFT processes that a Newtonian approximation could be assumed without noticing significant differences. The paste, though, may flow further in the simulations than in the experiments, due to the increase in the viscosity of the non-Newtonian paste at low shear rates after impacting onto the acceptor. In the model, the infinite-shear-rate viscosity was thus set to values within the range of 1–10 Pa·s for the thinner contents, as seen in Figure 2.

The dynamics of the transference were rather different if the presence of the acceptor interacted with the transferring paste or not. When the acceptor was placed at a distance around the paste layer thickness, the interaction was not neglectable: the acceptor slowed down the paste bell—and the bubble inside—when it interacted with the substrate, and its burst was thus prevented. In the case that the paste was ejected without an acceptor, only the rupture in the paste—leading to a burst spray or a cluster ejection—or the elastic confinement of the vaporized paste were possible—which turned into a non-transference regime. To study these conditions, a far acceptor was set to receive the ejected paste. On it, only a paste spray will be received, and no congruent structure will be deposited onto it [13].

### 4.1. Time-Resolved LIFT for High-Viscosity Pastes Regimes with No Acceptor

**Figure 5:**
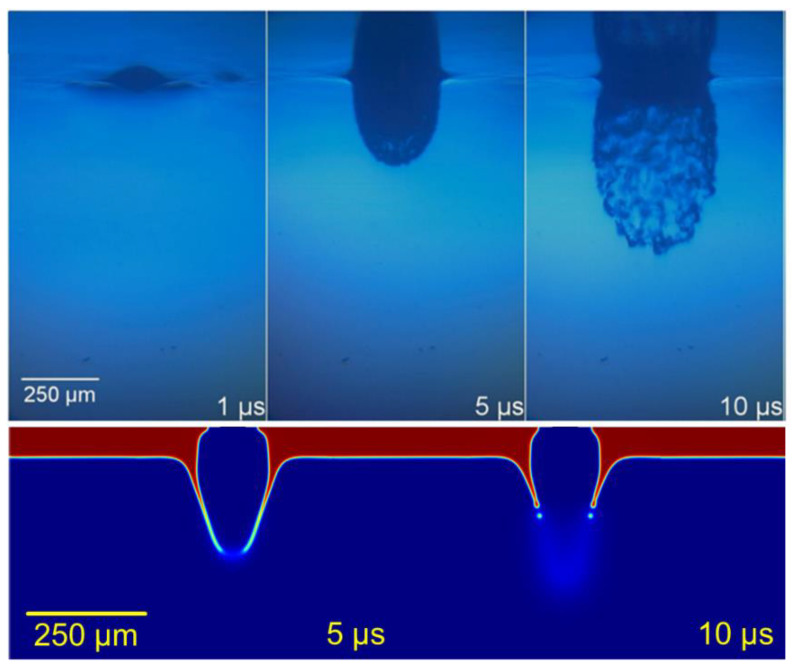
Explosive regime. Upper row adapted from [13], with PV17F paste. Lower row: simulation at 450 MPa at its maximum, for 60 μm of 2 Pa·s paste.

As in many other LIFT studies, pulse energy and donor fluid layer thickness were two strongly interrelated parameters. An increase in energy had similar effects to a decrease in the thickness of the transference layer. Thus, the high-viscosity LIFT of extremely thin layers had an intense bursting cone, whereas greater layers were susceptible to being deformed to a more intense extent before breaking (Figure 5).

**Figure 6:**
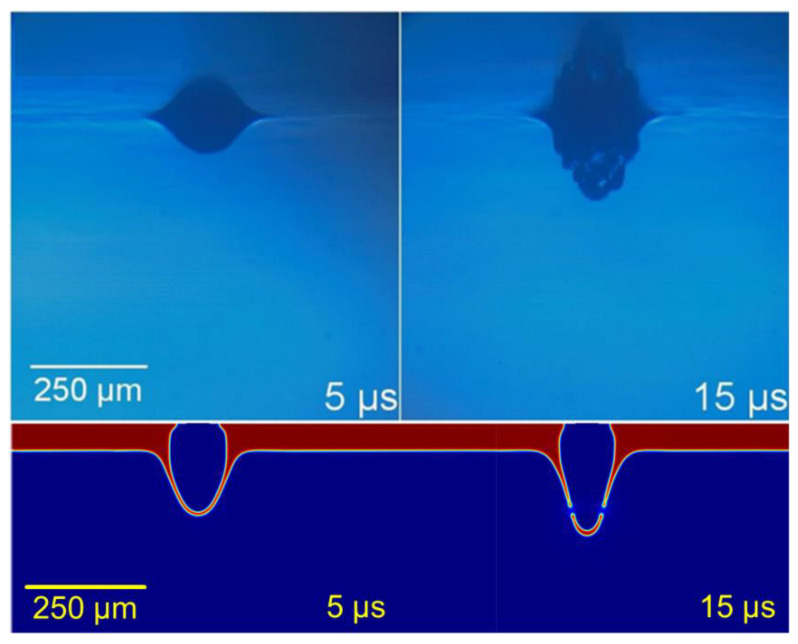
Cluster transference regime. Upper row adapted from [13], with PV17F paste. Lower row: simulation at 400 MPa at its maximum, for 60 μm of 2 Pa·s paste.

If the energy was slightly lower, and just above the transference threshold, a congruent voxel of paste could detach from the donor substrate and travel through the air. This led to a congruent dot-shaped transference, great in aspect ratio, but less usable when printing continuous lines because of the distance between the subsequent dots, which was often covered with paste spray (Figure 6).

**Figure 7:**
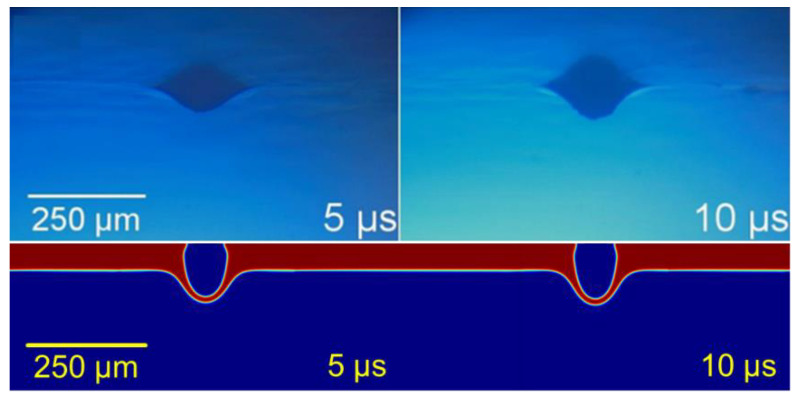
Non-transference regime. Upper row adapted from [13], with PV17F paste. Lower row: simulation at 300 MPa at its maximum, for 60 μm of 2 Pa·s paste.

Under an energy threshold that depended on the donor layer thickness, the viscous and inertial forces were strong enough to be overcome by the pressure in the bubble of vaporized paste. For low energy pulses, even though the paste vaporization took place, the bubble remained trapped in the paste and only an elastic recovery was observed, so no transference occurred onto the acceptor (Figure 7).

### 4.2. Time-Resolved LIFT for High-Viscosity Pastes Regimes Including an Acceptor

**Figure 8:**
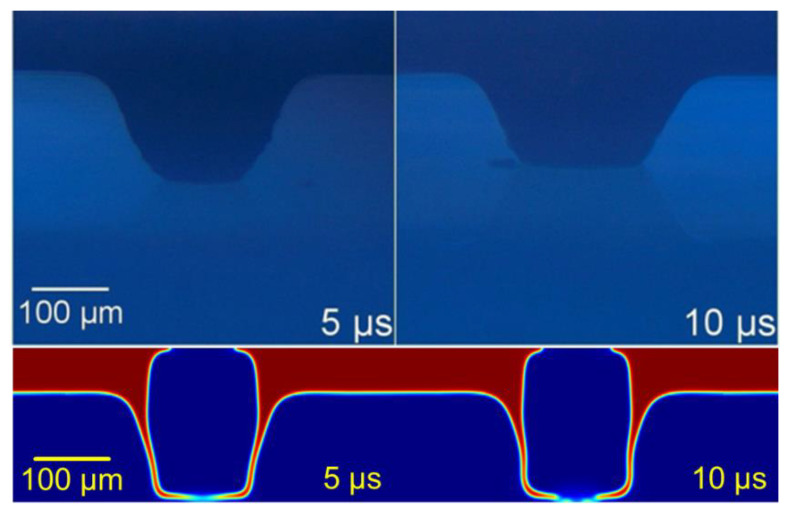
Stabilized column regime. Upper row adapted from [13], with PV17F paste. Lower row: simulation at 450 MPa at its maximum, for 60 μm of 2 Pa·s paste and a 140 μm gap.

The inclusion of an acceptor substrate modified the expansion of the bubble. Regarding its nature, the contact angle determined the adhesion between the paste and the substrate. The influence of the viscosity was so high that the contact angle did not play a principal role in the shape of the transferred paste. For this reason, an intermediate value of π/2 was set. In addition to the non-transference regime and the cluster transference, the explosive regime was now modified. This regime was formerly characterized by the disaggregation of the paste in small particles that broke down due to its colloid nature. Now, the paste was confined against a wall, so it stopped against the acceptor, and the expansion only continued in a radial direction (Figure 8).

For lower energies, the bubble expanded to the acceptor, but the pressure did not break the column radially, so a stable columnar structure was set between the donor and the acceptor substrates. The stability of this structure was determined by the paste viscosity, the gap between the substrates, and the contact angle between the paste and the acceptor; the higher the viscosity or the lower the gap is, the easier the column may be established.

### 4.3. Regime Comparison in LIFT for High-Viscosity Pastes

**Figure 9:**
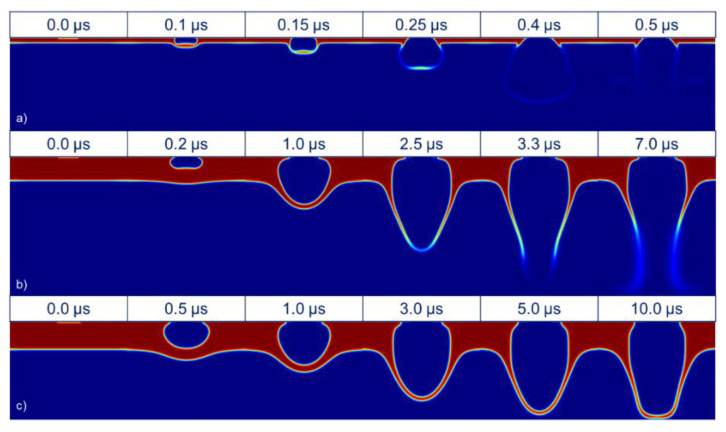
Explosive regime (**a**) simulation of a 2 Pa·s paste for a low thickness (12 μm) with a bubble pressure of 300 MPa, (**b**) simulation of 2 Pa·s paste for intermediate thickness (60 μm) with a bubble pressure of 500 MPa, and columnar regime, and (**c**) simulation of 2 Pa·s paste for intermediate thickness (60 μm) with a bubble pressure of 400 MPa and a gap of 160 μm.

Some effects were observed during the experimental transference of high-viscosity metallic pastes. Once the model was validated through a comparison with direct observation images, the three main regimes could be replicated in the model. When the acceptor was far enough (typically more than twice the fluid layer thickness), the paste burst before touching the acceptor substrate, and only a paste spray reached it (Figure 9a,b). On the contrary, when an acceptor substrate was put close enough to the donor, the expansion of the paste slowed, and the burst was prevented, so a stable column could be established between both substrates (Figure 9c).

**Figure 10:**
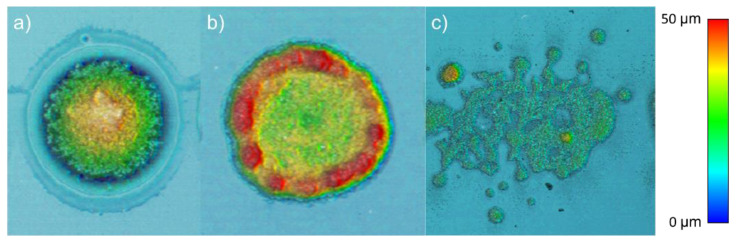
Dot transferred with energy values just over the threshold (**a**), crowned structure after breaking a stable column established between both substrates (**b**), and paste spray onto the acceptor substrate (**c**), and from PV19B 5 wt. % organic solvent added paste.

Experimentally, low values of energy did not cause the transference of the paste. Over the threshold, the paste first touched the acceptor in the shape of a dot, as in Figure 9c. An example of this transference is shown in Figure 10a. When the pulse energy was higher, the column grew wider, with a much bigger hollow cavity inside, as in the simulation in Figure 8, which led to a crowned dot (Figure 10b). The explosive regime, modeled in Figure 9a,b, led to spray structures (Figure 10c).

## 5. Results

**Figure 11:**
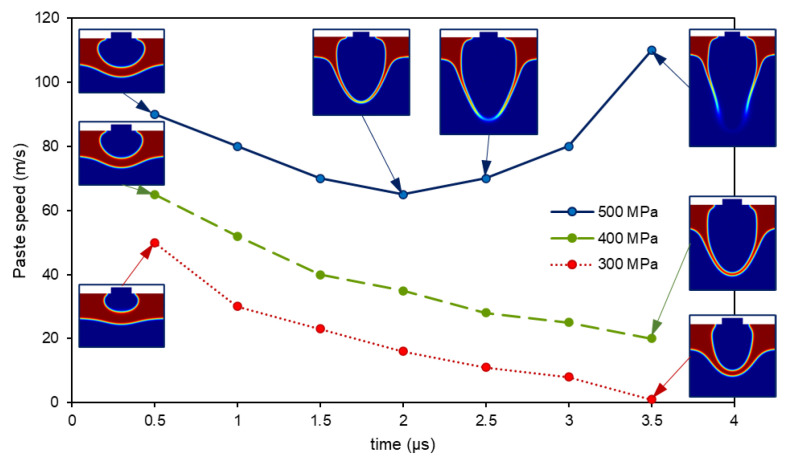
Paste speed versus time for a 60 μm paste layer and an initial pressure of 300 MPa (dotted red line), 400 MPa (dashed green line), and 500 MPa (solid blue line). The burst of the paste can be seen for the last case, in which the speed raises after slowing down.

In Figure 11, the average speed of the paste over the symmetry axis (r = 0) is plotted for three different pressures. The analysis of the simulations for the cases without an acceptor substrate in which the paste did not burst led to a common speed pattern: the paste and the air inside the bubble slowed down as the expansion happened. When the structure broke up, the new behavior was pointed out: the cohesive forces of the paste did not beat the inertial ones, so the fluid throttled up again and burst into a paste spray, as seen in Figure 11 (500 MPa, blue solid line). On the contrary, lower values of pressure were not enough to break the paste, and it slowed down until it stopped (Figure 11, 400 and 300 MPa, red dashed and green dotted lines, respectively). These phenomena have already been observed by direct image acquisition [13].

**Figure 12:**
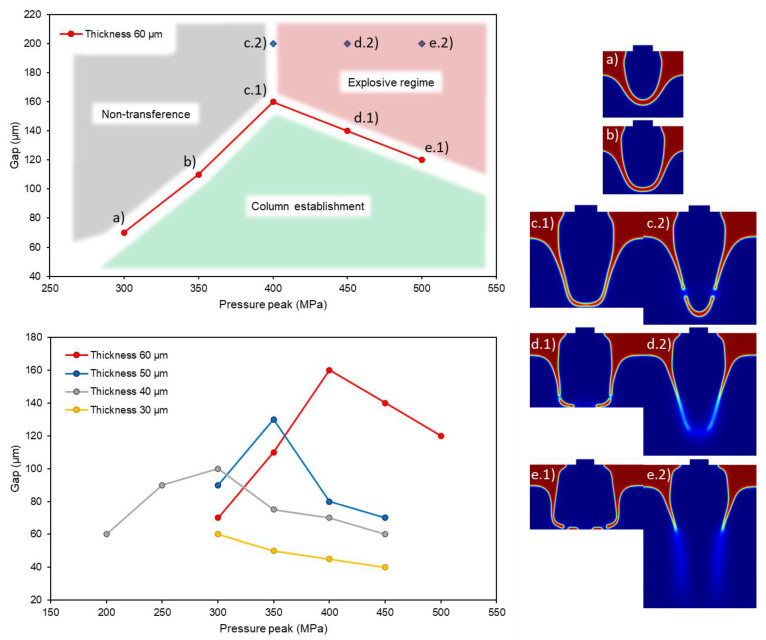
Study of the maximum gap for the formation of a columnar structure. Analysis for a single fluid thickness (60 μm) in which the three main regimes are distinguished: non-transference, column formation and explosive regime (upper graph), and comparison for different thicknesses (30 to 60 μm) in which the maximum is displaced to lower values of gap and pressure (lower graph).

Not only the burst of the paste can be predicted, but also the maximum distance at which the transference is effective. The formation of a stable column depends on both the pressure due to the laser interaction and the gap between the donor and the acceptor substrates. For the values at which the paste did not burst, a gap greater than the maximum expansion of the paste led to a non-transference regime, whereas a lesser gap made the paste touch the acceptor (Figure 12, upper chart, left from the apex; a,b). If the pressure was high enough to make the paste burst, placing the acceptor close made the expansion stop and prevented the paste from exploding, as seen in the pair comparisons Figure 12, c–e. In these cases, the bursting of the already established column occurred radially for high pressures. The limit case—the apex—happened when the paste detached without bursting. This case corresponds to the point c.2 in Figure 12, upper chart, and its transference is shown in Figure 12, c.2.

Given the paste thickness (60 μm in Figure 12, upper chart), there are three distinguishable regimes, where the dots represent the extreme cases of columnar structures (maximum distance before stopping (a and b) and maximum distance for preventing the burst (c, d, and e) depending on the case). Considering a fixed gap, the formation of a column only occurred in a range of pressures. If the pulse was not intense enough, the paste did not touch the acceptor and the transference did not take place (non-transference regime). On the contrary, over a maximum value, the column formed but the pressure inside was high enough to make it burst and spray onto the acceptor. For even greater values, the column did not even form, and just the spray travelled towards the acceptor (explosive regime). When the substrates were too spaced, there were no conditions for the paste to stretch and touch without breaking, so the transference process can be considered “without acceptor”, as it did not influence the dynamics of the paste. This study was done by varying the thickness of the paste, and it was then observed that the thinner the paste film, the lower the value of pressure to make the paste burst. In Figure 12, lower chart, 30 μm thickness line (yellow solid line), the apex is not even simulated in this work.

However, considering a gap smaller than the thickness, the conditions for the formation of a column widened despite it not being a proper cluster of paste, but a big area on touch. The lower limit of the pressure now showed the non-transference point as before, and the higher limit defined the radial burst point. It is not typical to set a gap that is smaller than the thickness because of the technical limitations of the placement equipment. The acquiring images of the experiments are much more difficult to carry out with small gaps, as the light must cross trough the groove between the paste and the acceptor in shadowgraphy.

**Figure 13:**
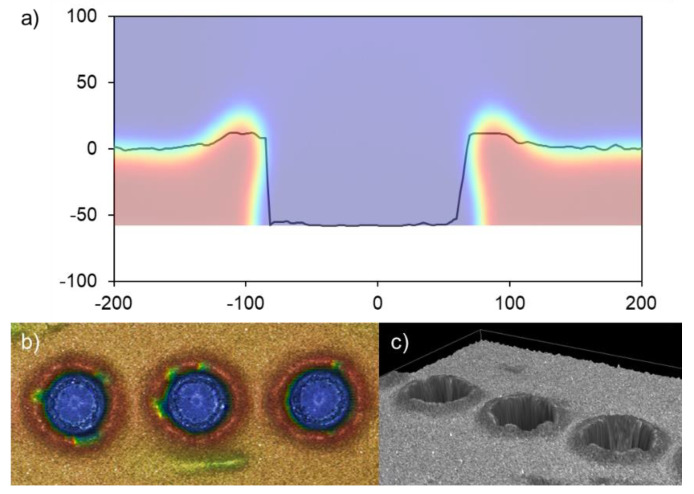
Annular bulge shaping in the donor after the paste burst for the explosive regime. Confocal profile superposed with a FEM-CFD simulation at a long time—without further evolution—(**a**), a confocal top-view image (**b**), and a confocal 3D view (**c**) are displayed, done with PV19B paste.

In addition, apart from the different transference regimes, several phenomena can be explained through the proposed model. For instance, the transference sets a hollow annular bulge morphology in the donor due to the emptying of the donor layer, as observed in the model. The paste does not flow back and refills the hollow dot because of its high viscosity. The comparison is shown in Figure 13, along with a profile measurement, a top-view, and a 3D view acquired through confocal microscopy.

## 6. Conclusions

In this work, the transference mechanisms of LIFT for high-viscosity fluids of silver microparticle pastes have been studied. These fluids are mainly used in the photovoltaics industry, but their rheology is shared by some high-viscosity biofluids. The different regimes observed using time-resolved shadowgraphy have been replicated in a numerical model implemented in COMSOL: non-transference (with its elastic recovery), cluster transference, bursting cone, and stable column. These results have been compared with the images already published. The model allowed for gaining insight into the physics of the transference in the case of LIFT for high-viscosity pastes.

In the model, the motion of the fluid is induced through an inlet, which permits some highly pressurized air to enter the domain resembling the vaporization of the paste after the interaction with the laser pulse. The shape of the simulated structures agrees with the experimental images in the bibliography, as well as with the behavior of the paste remaining behind, already described in the bibliography.

The analysis of the simulations shows that the paste slows down during its transference. Low pressures make the paste stop before reaching the acceptor, and when the pressure becomes equal to the ambient pressure the bubble will keep trapped—in the non-transference regime. The interposition of the acceptor at a distance at which it interacts with the paste before stopping will form a stable column [23]. If the gap is more than three times the thickness of the paste, the remaining pressure inside the paste structure will make it burst, leading to its rupture in the shape of a cluster or a spray.

In the simulations, it is observed that the paste and the air inside throttle up after the burst of the paste structure. The maximum length of the bell-like structure before this acceleration equals the maximum distance at which the acceptor substrate can be placed to guarantee the transference—slightly below three times the thickness of the paste layer. Gaps above this threshold value will lead to a spray bursting regime [24]. The variation of the paste layer thickness shows that the maximum gap decreases when the thickness does. On the contrary, small gaps ease the establishment of a column between an energy pulse limits: too small values do not move the paste to make it touch the acceptor, and extremely high ones will make it burst even after touching.

## Data Availability

The data presented in this study may be available on request from the corresponding author. The data are not publicly available because the model is still under development for further studies.
